# Implementation of workflow engine technology to deliver basic clinical decision support functionality

**DOI:** 10.1186/1471-2288-11-43

**Published:** 2011-04-10

**Authors:** Vojtech Huser, Luke V Rasmussen, Ryan Oberg, Justin B Starren

**Affiliations:** 1 Biomedical Informatics Research Center, Marshfield Clinic, Marshfield, WI, USA; 2 Morgridge Institute for Research, Madison, WI, USA; 3 Division of Biomedical Informatics, Department of Preventive Medicine, Feinberg School of Medicine, Northwestern University, Evanston, IL, USA

## Abstract

**Background:**

Workflow engine technology represents a new class of software with the ability to graphically model step-based knowledge. We present application of this novel technology to the domain of clinical decision support. Successful implementation of decision support within an electronic health record (EHR) remains an unsolved research challenge. Previous research efforts were mostly based on healthcare-specific representation standards and execution engines and did not reach wide adoption. We focus on two challenges in decision support systems: the ability to test decision logic on retrospective data prior prospective deployment and the challenge of user-friendly representation of clinical logic.

**Results:**

We present our implementation of a workflow engine technology that addresses the two above-described challenges in delivering clinical decision support. Our system is based on a cross-industry standard of XML (extensible markup language) process definition language (XPDL). The core components of the system are a workflow editor for modeling clinical scenarios and a workflow engine for execution of those scenarios. We demonstrate, with an open-source and publicly available workflow suite, that clinical decision support logic can be executed on retrospective data. The same flowchart-based representation can also function in a prospective mode where the system can be integrated with an EHR system and respond to real-time clinical events. We limit the scope of our implementation to decision support content generation (which can be EHR system vendor independent). We do not focus on supporting complex decision support content delivery mechanisms due to lack of standardization of EHR systems in this area. We present results of our evaluation of the flowchart-based graphical notation as well as architectural evaluation of our implementation using an established evaluation framework for clinical decision support architecture.

**Conclusions:**

We describe an implementation of a free workflow technology software suite (available at http://code.google.com/p/healthflow) and its application in the domain of clinical decision support. Our implementation seamlessly supports clinical logic testing on retrospective data and offers a user-friendly knowledge representation paradigm. With the presented software implementation, we demonstrate that workflow engine technology can provide a decision support platform which evaluates well against an established clinical decision support architecture evaluation framework. Due to cross-industry usage of workflow engine technology, we can expect significant future functionality enhancements that will further improve the technology's capacity to serve as a clinical decision support platform.

## Background

Currently, there is a strong interest in improving decision support systems (DSS) [1]. Despite several decades of effort, we have been unable to develop DSS platforms that would gain wide adoption [2]. Some solutions are embedded in a proprietary system or are tied to a particular electronic health record (EHR) vendor which limits their adoption. Other solutions often introduce a healthcare-specific representation format and healthcare-specific execution engines, whereas past experience shows that successful healthcare solutions often rely on cross-industry standards. Finally, easy authoring or easy review of DSS logic still remains a considerable challenge [3]. We present our implementation of a workflow engine technology [4] which addresses two current challenges of DSSs.

The first challenge is the ability to evaluate DSS modules prior to deployment. For seamless testing and deployment, it is beneficial to be able to easily switch the execution of a DSS module from prospective to retrospective mode. Traditionally, this problem has been solved by two approaches, both of them sub-optimal and requiring additional resources. The first approach involves deployment of the module at a pilot site prior to enterprise-wide deployment and fine tuning the logic directly within the deployment environment. The second approach is a separate side-project for each deployed module which extensively analyses the possible impact of the intended DSS module. This separate side-testing usually involves a separate DSS logic representation for such retrospective testing (compared to the deployment prospective version of the logic).

The second challenge is the ability of non-programming clinicians (as recipients, reviewers, maintainers, or authors of decision support) to understand and manipulate the logic of a given DSS module. From a perspective of non-programmers, it can also be described as a "black-box phenomenon." A clinician who cannot review in detail the logic of a given decision support system may be reluctant to adopt a system that he can not fully understand.

We were able to address the two above-mentioned challenges with a system called *HealthFlow*, which is an implementation of a workflow engine in the context of an EHR system. This software category article aims to provide implementation details for informaticians and champion clinicians at healthcare organizations that may be considering workflow engine technology to enhance their decision support functionality. We use the term *clinical logic *to encompass not only decision support problems, but also knowledge representation for domains of quality improvement and clinical research alerts [5]. The HealthFlow project is an effort to utilize a workflow editor and a cross-industry process definition standard to represent clinical logic and to use a workflow engine to execute such logic. Key objectives of the HealthFlow project are: (1) the ability to switch seamlessly from retrospective execution mode for prior-deployment testing to prospective mode; (2) the ability of non-programmers to review the executable logic in a user-friendly fashion (graphical, step-based flowcharts); and (3) the interoperability of the encoded executable logic across different healthcare institutions. The HealthFlow system described in this article consists of two components that share a set of common characteristics. We use the term *RetroGuide *for the retrospective mode of operation, and our initial work with workflow technology focused on modeling retrospective and analytical processes is presented elsewhere [6] (a comprehensive set of our desired functional requirements for a healthcare process modeling platform is published separately [7]). For the prospective component of the system, we use the term *FlowGuide*, and it was developed later as a distinct component within the HealthFlow project. The functional specification for the prospective component did not stem from a fixed set of initial requirements, but instead were an effort to maximize the use of functionality already included in a workflow technology suite.

Several prior studies report the use of workflow technology (WT) in healthcare, and we briefly survey some of these studies. Emanuele [8] presents the use of WT to improve infection control and proposed a term *workflow-enabled EHR system*, which can communicate bi-directionally with a Workflow Management System (WfMS); e.g., send EHR event notifications to the workflow engine and display in the EHR system tasks and alerts generated by the workflow engine. Quaglini et al [9] piloted the use of the Oracle workflow software suite to implement a stroke guideline. Their project used a non-standard and proprietary process definition language; the workflow engine generated tasks, and alerts were delivered to clinicians via email. Peleg [10] discusses the close relationship of current workflow engines with clinical decision support engines, and in collaboration with Mulyar [11,12], compared existing guideline representation formats with workflow process definition standards using workflow patterns [13]. Finally, Haux [14] describes a commercial EHR system that tightly implements a workflow engine with clinical care information technology (IT) systems to enable advanced customization of many EHR functions to address local needs and utilize location-specific resources. The implementation presented in this article extends this prior work and proposes a prospective as well as retrospective operation mode of utilizing a workflow engine. Unlike previous implementation, it is also a solution that relies on established workflow technology standards rather then proprietary process definition languages. A later subsection of this article (architectural evaluation) further compares and analyzes workflow technology-based approach to decision support in comparison to other decision support platform using an architectural evaluation model [15]. In the following section, we present the architecture overview of our WT implementation, typical usage phases, how the system interfaces with available clinical data and healthcare environment, and a use case example.

## Implementation

### Overview of the architecture

HealthFlow system is an implementation of a workflow management system (WfMS) [4]. Such a system offers the ability to model a process as a graphical flowchart in a workflow editor and execute such process in a workflow engine. A workflow process would contain steps involved in a given decision support problem (e.g., hypertension screening logic). We prefer to use the term *scenario *as a clinician-friendly synonym to a workflow process in order to better communicate the clinical context of a given workflow process definition.

**Figure 1:**
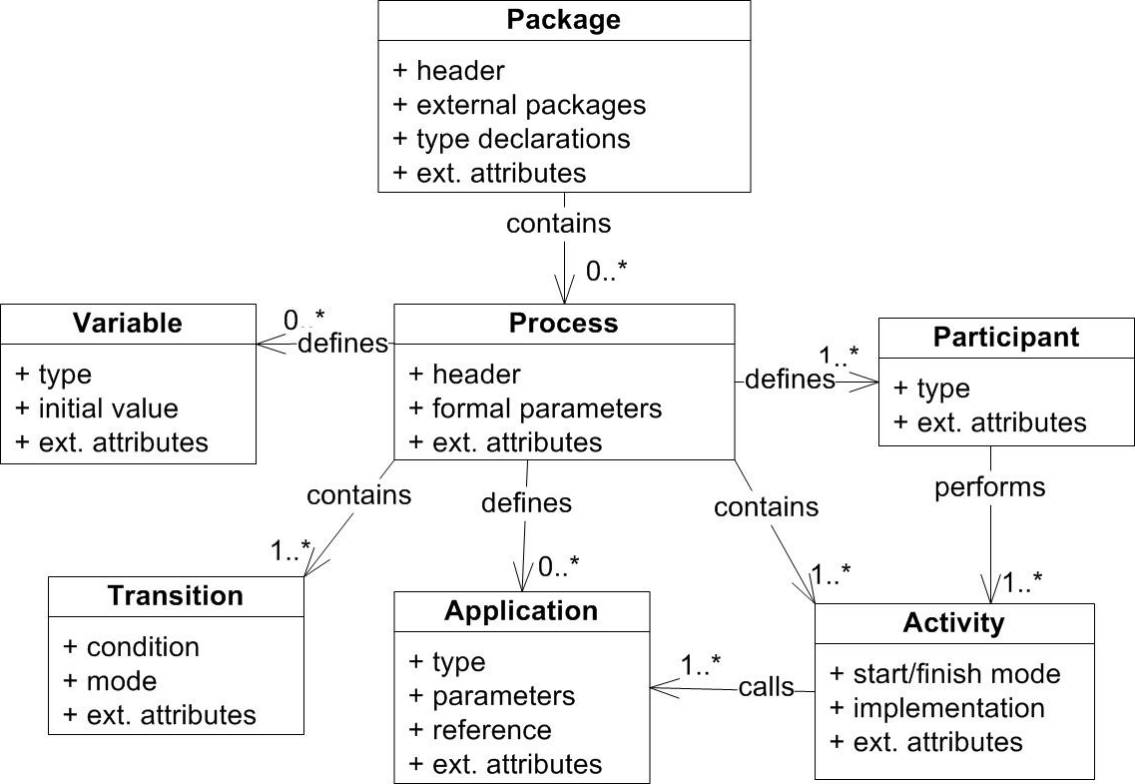
**XPDL standard**. UML Class Diagram showing key XML Process Definition Language (XPDL) classes. Activities and transitions are key components which form a process definition. Additional process elements are applications, workflow variables and participants.

We currently use Together Workflow Editor http://sourceforge.net/projects/jawe as our main editor for viewing and creating executable scenarios. HealthFlow utilizes a standard XML Process Definition Language (XPDL) which is a standard defined and maintained by Workflow Management Coalition (WfMC; http://www.wfmc.org/xpdl.html). Adherence to this standard avoids vendor lock-in problems (ability to switch to workflow suite from a different vendor). It also enables inter-institutional sharing of the modeled scenarios. Figure 1 shows a high-level class diagram of the XPDL standard with the root element of a workflow package containing individual process definitions. Key components of each process definition are applications, participants, activities, transitions, and variables [16]. Additional diagrams of the XPDL schema are presented in [Additional file 1].

For scenario execution, we use Together Workflow Engine http://sourceforge.net/projects/sharkwf. Our existing implementation structurally relies on an open source workflow suite; however, it can also be implemented on more feature-rich, commercial, XPDL-compliant workflow suites (e.g., Tibco business process management platform from Tibco software (Palo Alto, CA, USA) or Fujitsu Interstage platform). We chose the XPDL standard over other workflow definition languages (e.g., Business Process Execution language (BPEL), [17]) or Business Process Modeling Notation (BPMN), [18]) because it has the widest adoption among the workflow system vendors [19]. Another advantage of the XPDL standard (in contrast to BPEL) was its ability to provide a corresponding graphical flowchart-like version of a processes definition in addition to a code-like version.

To provide an interface to clinical data and the EHR system as well as some other custom functionality, HealthFlow extends the workflow engine and editor with a set of small and modular *external applications *(EAs). The majority of the logic of the clinical scenarios and the key strengths of our implementation lie in using XPDL graphical flowchart constructs. External applications are used only when XPDL constructs are insufficient or for interfacing with other systems. In our current implementation, these lightweight and modular external applications are developed in Java, but other modalities are possible with most workflow management systems, such as a web service, a JavaScript script, or executables programmed in other programming languages. Also, these applications currently operate on a native HealthFlow event model, but it is possible to create versions of those applications that operate on other event models; for example event models and data structures of a vendor based EHR system, openMRS (Open Medical Records System, [20]), or HL7's vMR model (Virtual Medical Record [21]). For a detailed description and purpose of an event model, we refer the reader to Huff et al.'s work [22]. In pilot experiments, we have successfully demonstrated HealthFlow's ability to operate on an event model of version 1.3 of i2b2 (as represented in the i2b2 OBSERVATION_FACT table schema). i2b2 [23,24] stands for Informatics for Integrating Biology and the Bedside and it is an emerging platform for storing and querying EHR data, developed at Partners HealthCare with a grant from the National Institute of Health, USA. This proves that HealthFlow capabilities can be easily adopted by sites that converted and imported their data into an i2b2 repository. We use HealthFlow event model because it offers a more elaborate event structure than i2b2 native model (version 1.3 of i2b2). In HealthFlow event model certain common event characteristics are represented as native event data components (e.g., event type and event subtype), instead of using i2b2's event attributes structure. We further comment on the relationship of HealthFlow and i2b2 in the discussion section.

Similar to the event model, where HealthFlow EAs access EHR data, for final delivery of generated decision support content, it is important to provide similar interface via EAs to the host EHR system. Examples include the ability to modify or respond to action within EHR screens handling computerized order entry, problem list maintenance, results review, and treatment planning (referral and prescribing). In our work, we focused mostly on generation of decision support content and non-standardized basic delivery of this content at a single institution (e.g., daily batched delivery of generated content to a care coordinator role rather than scenarios with fully-developed and sophisticated alert delivery and usability logic). Ability to integrate into the EHR depends on the decision support platform but also on the host EHR. Peleg [10] defines this as a third level of decision support integration, and Emanuele and Koetter [8] use the term "workflow-engine enabled" EHR system when the EHR can communicate with a workflow engine by sending pertinent healthcare event data to the engine and conversely receive tasks generated by the engine.

### Multiple complexity levels

Various decision support solutions (e.g., Arden Syntax [25], GLIF (Guideline Interchange Format, [3]), EON [26], ProForma [27]) have addressed with various degrees of success many challenges in DSS [10]. We would like to discuss two factors that we consider important: (1) a given DSS platform can support multiple levels of complexity on a complexity-functionality curve; and (2) a DSS platform provides a flexible interface to the outside-world. A common theme in DSS, since the days of Arden Syntax's curly braces problem [28], is the fact that a DSS platform must interface with external systems (e.g., EHR system or/and Clinical Data Repository). Dealing with external complexity may in fact require significantly more expertise then constructing the clinical logic itself. To achieve balance between complexity and functionality, we have designed the HealthFlow system to have three different levels of external complexity (basic, advanced, and ultimate). We refer to these three levels as HealthFlow *usage levels *because they entail multiple aspects. For example, a simple decision support problem can be fully captured on a basic usage level (e.g., obtain one most recent laboratory result for a single test, compare it to a critical threshold, and alert); whereas, a more complex logic operating with multiple parameters that may interact with each other, may require advanced or ultimate HealthFlow usage level. The first aspect of the usage level is the complexity and configurability of the external applications employed by the scenario flowchart. The second usage level aspect is the overall look of the scenario flowchart (e.g., basic usage level flowcharts [or flowchart steps] are easier to understand for non-programmers than flowcharts at the advanced or ultimate usage level). We discuss the three usage levels in greater detail in a later sub-section describing the external applications.

### Three HealthFlow usage phases

**Figure 2:**
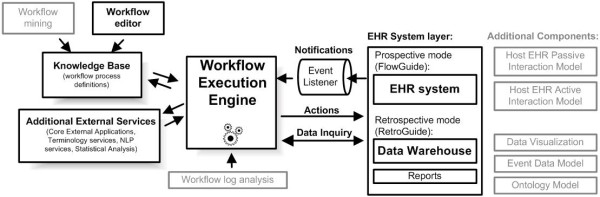
**Implementation architecture**. High-level diagram of the architecture of our implementation of the workflow engine (HealthFlow system). The key components are shown in bold. The workflow engine is bi-directionally connected to an EHR system layer. The left part shows a scenario knowledge base, authored within a workflow editor, and external services which include core HealthFlow external applications.

The use of HealthFlow can be divided into three phases; (1) scenario creation, (2) scenario testing (RetroGuide), and (3) scenario deployment (FlowGuide). In most cases, phases 1 and 2 are repeated iteratively until the resulting scenario and HealthFlow-generated reports fully address the knowledge representation problem at hand. Depending on the nature of the project, the phase 2 scenario testing version may be deployed as a prospective module with no or small modifications. This ability directly addresses the first challenge we described in the introduction (ability to test clinical logic on retrospective data). In order to understand the relationship of the retrospective and prospective mode of HealthFlow operation, we must first explain the concept of a current temporal position in the EHR record. In *retrospective mode (RetroGuide) *the scenario consumes all available EHR events; it does not wait for an event to happen, but instead searches for it beyond the current temporal position. A data warehouse (or other comparable clinical data repository entity) is a primary component used in this mode, as it provides an optimized platform for searching through all historical clinical data. A given step in the scenario flowchart simply retrieves retrospective EHR data. For example, the step may contain logic to "find date of the next outpatient visit to pediatrics after discharge from hospital". In *prospective mode (FlowGuide)*, however, the "future-oriented" scenario flowchart steps are switched to wait for a particular event to arrive via event listener. In this mode, the engine communicates directly with the EHR system in real-time. To illustrate this on the previous example of pediatric visit, the workflow engine would maintain a status of a scenario instance, which would be in paused mode, until a pediatric visit would happen. Figure 2 shows the overview of the architecture and depicts the two modes of operation. It shows that the FlowGuide component primarily interacts with an EHR system and point-of-care, whereas the RetroGuide component works with a data warehouse layer, and scenario conclusions (proposed prospective actions) are merely documented in a retrospective execution report and not sent to the point-of-care. Figure 2 also shows additional components of the architecture, such as the knowledge base consisting of process definitions (scenarios) and workflow editor. The set of HealthFlow modular external applications (EAs) is included in the additional external services box. The figure also includes (in gray) additional architectural components such as the event model and the ability to analyze workflow logs or obtain scenario definitions using workflow process mining techniques [29].

Before describing the individual phases, we need to more precisely define a HealthFlow scenario. A *scenario*, which is in workflow technology terms a fully executable workflow process definition, consists of two key layers: a graphical flowchart layer and a hidden code layer. The *flowchart layer *can be created and reviewed by users with limited programming expertise (e.g., champion clinicians or other non-expert requestors of a clinical logic module such as administrative and management level healthcare personnel or researchers). The *code layer *is hidden behind the nodes and arrows of the flowchart and contains references to modular applications that can (1) obtain EHR data (or listen/wait for them), (2) provide various analytical functions using any external technology, or (3) perform an action within an EHR system at the point-of-care (or document such action within a report when in retrospective mode). This hidden code essentially utilizes HealthFlow EAs, or it can call any external computer application or a web service. Finally, a HealthFlow scenario has yet an additional *variable layer *where relevant information can be passed between flowchart steps (e.g, average systolic blood pressure at age 60-65). We will describe each of the three HealthFlow phases in detail.

#### First phase: Scenario Development

**Figure 3:**
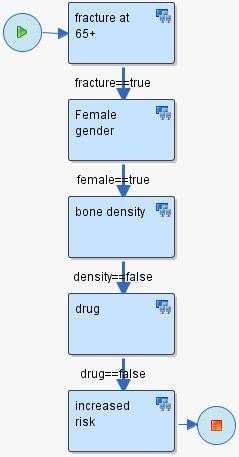
**Retrospective scenario flowchart example**. Example of a RetroGuide osteoporosis scenario flowchart with analytical steps. This flowchart can operate on retrospective data because it does not require interaction with a clinician. All steps of the flowchart involve searching for a prior EHR event. The following logic is represented: (1) In patients with a fracture after age 65; (2) ensure female gender; (3) ensure that patient did not have a bone mineral density test performed; (4) ensure that no osteoporosis prevention drug has ever been prescribed; and (4) conclude that the patient is at increased risk for fractures.

The first phase of scenario development involves the creation of a sequence of steps, relying on HealthFlow's external applications. Each step is modeled as a node in a flowchart. Each node may contain the execution of one or more EAs. Arcs connecting the nodes represent the flow of logic. See figure 3 for an example scenario. Six additional scenario examples can also be found in [Additional file 2]. The graphical nature of a HealthFlow scenario facilitates collaboration between a (1) *requestor *(usually a clinician, but it can also be a researcher, administrator, or other domain expert) who develops the scenario using the graphical flowchart and the textual description fields that specify what each node or arc should perform, and the (2) *collaborating knowledge engineer*, knowledgeable about EAs, who makes the scenario executable by filling in the necessary computer-interpretable components.

#### Second phase: Scenario Testing (RetroGuide)

During the second scenario testing (RetroGuide) phase, the model is executed by the workflow engine using retrospective data from a set of patients (testing cohort). The scenario testing phase can be divided into two major steps: actual execution, and a reports review step. During the *execution *step, the workflow engine uses a single patient modeling and execution approach, where the scenario is run for each patient as an individual. Several reports are built during the execution step, capturing the execution trace of the scenario for each executed patient from the testing cohort. In addition to a single patient execution trace, data views are also provided to enable population view of the execution. Optional custom reports can also be built, documenting patients that satisfied certain combinations of steps or conditions (conclusion steps). The execution by default proceeds forward in time, and the concept of *current position *in the chart is important. The process is analogous to a human research abstractor performing manual chart review. The process is best understood by envisioning a robot that is browsing a chronologically ordered series of coded clinical events found in an EHR. The robot (RetroGuide) hypothetically starts at the first EHR event and advances through the record according to the flowchart step-wise instructions.

The second step of *reports review *involves the analysis of how well the HealthFlow scenario addresses the clinical problem or logic at hand. This is achieved by using HealthFlow data visualization components (see figure 2). Three main data views are available: summary report, detailed report, and individual patient view. HealthFlow *summary report *lists cohort-based statistical results, e.g., "How many cohort patients satisfied a particular scenario step, branch or condition?" The second, *detailed report *is patient-centered and offers additional details about scenario execution, e.g., exact values, timestamps and codes for individual EHR events investigated by the scenario. Finally, the third report offered by HealthFlow, the *individual patient view *(IPV), lists all EHR events for a particular patient. The review of selected patients within the detailed report and individual patient view is the basis for iterative improvements to the scenario logic. Several examples of scenario flowcharts and reports can be found on the HealthFlow project website http://healthcareworkflow.wordpress.com.

During retrospective testing, several aspects of the scenario are evaluated which are mainly oriented towards whether a meaningful decision support content is generated out of a proposed scenario logic:

(1) *What percentage of the initial testing cohort has an action/intervention alert generated?*

This evaluation aspect is based on the assumption that a given new scenario checks certain clinical aspects of a patient which results in no action or intervention in compliant patients or some new action in non-compliant patients. In the first compliant case, the clinician's work is not changed and no interruptions occur. In the second non-compliant case, the clinician's work may be affected by one or several types of new actions. For example, new relevant information is being passively displayed, or the user is interrupted with a new data entry or choice selection dialog. In other words, if the scenario logic checks a quality improvement initiative and the non-compliant percentage of patients is too high (e.g., more then 50%), the new decision support content could be too aggressive, and other means of intervening may be chosen (e.g., quarterly educational intervention supported by peer-comparison and retrospective data review, rather then too frequent, point-of-care decision support). Similarly, if the number of affected patients is too low (e.g., less then 3%), it may not be cost effective to justify creation and costly enterprise maintenance of a new decision support content for this problem. However, this also depends on the severity of the clinical consequences in a non-compliant patient. Iterative review and changes to the trigger criteria, clinical logic thresholds, or number and types of possible output actions during this second RetroGuide phase can result in achieving an optimum balance between the projected impact of the new decision support module on clinicians, necessary information campaign about the new module, and significance of the new module's clinical domain.

(2) *Does the scenario logic perform similarly on additional testing cohorts (e.g., other geographic regions, different physician groups)?*

The initial testing is usually done in a cohort suggested by the scenario requestor(s) (e.g., patients from a given clinic with certain trigger criteria). After basic scenario logic fine tuning, an integral part of the retrospective testing phase is execution on cohorts from other clinics or on an enterprise-wide cohort, to see whether the same logic can be deployed enterprise-wide, or whether the new logic will require more complex local customizations (e.g., same logic applied only in selected sub-populations [physician groups, regions, or patient groups]) or several versions of the logic with slight modifications for different contexts. In some implemented scenarios, retrospective testing in additional cohorts significantly influences the final logic, and the scenario authors are surprised with results of this testing and variability and number of possible data patterns in different populations. In such cases, there could be tens of iterations between scenario modifications and testing.

(3) *Do additional exclusion criteria need to be added, based on review of patients affected by the new logic?*

For example, during retrospective testing of the newly proposed very high cholesterol patient scenario (patients with recent low-density lipoprotein [LDL] lab result of 190 mg/dL or more), we additionally excluded patients coming for oncology and/or optometry appointments based on the requestors clinical consensus. It is important to also note, that while review of retrospective results provides useful inputs, it may not cover all possible situations depending on the size of the retrospective cohort. It complements, but not eliminates, evaluation of the later prospective scenario deployment.

The functionality of the retrospective RetroGuide component of our workflow engine implementation can also be used as a standalone tool for flowchart-driven data analysis and is closely related to many cohort size estimation query tools [6]. However, the ability to use the same flowchart representation paradigm for retrospective as well as prospective execution mode distinguishes our implementation from many other research efforts. Additional examples and descriptions of the second RetroGuide phase can be found in published case studies on Hodgkin's lymphoma [30], hypertension [31], inpatient glucose control protocol [32], hepatitis C treatment and adverse drug events after use of narcotics [33], and osteoporosis and cholesterol control [34].

#### Third phase: Scenario Deployment (FlowGuide)

The third phase of scenario deployment (FlowGuide) uses the same framework as the retrospective RetroGuide phase, with a few exceptions. FlowGuide prospective scenario may be created from scratch (very uncommon) or can be based on a retrospective RetroGuide scenario (most frequent). We will focus our description on the second case and also explain what changes occur when a retrospective scenario is made to run prospectively. A FlowGuide scenario consists of five types of possible flowchart steps: trigger, background, analytical, listen, and action nodes. Two of the types, background and analytical nodes, are also present in retrospective RetroGuide scenarios. *Trigger steps *start the scenario. For example, a visit to rheumatology is scheduled for today or a surgical report is filed: there can be one or multiple trigger steps. *Background *steps find past events in the EHR (at runtime). For example, find earliest instance of rheumatoid arthritis diagnosis or find latest pharmacy record (prescription, renewal, or discontinuation) for a biologic agent such as infliximab. *Analytical *steps manipulate data obtained via previous flowchart steps. For example, based on average systolic blood pressure value, determine follow-up frequency criterion (every year or every two years). *Listen *steps wait for a given EHR event to occur in real time. For example, wait for a discharge event in a patient previously triggered by a surgical report creation. Listen steps share the same event data model with background steps. During retrospective-to-prospective scenario translation, listen steps are created from background steps by changing the mode from retrospective search scope (search for a specific past EHR event in a given patient) to waiting, prospective scope (wait until a specific event occurs to a given patient). Finally, *action steps *perform actions within a given EHR system (or other related system, e.g., Clinical Trial Management System). Actions may involve updating a report or registry, sending an email, generating an alert, creation of an order, or order cancellation. Full range of previously identified intervention types (notify, log, provide defaults, show guidelines, etc.) is supported with proper interface support of the intervention target system [35,36]. During retrospective-to-prospective scenario translation, action steps are often created from conclusion steps by changing the action from 'retrospective report conclusion creation' to a particular prospective action. Although we emphasize the ability to test a given clinical logic retrospectively, it is important to note that not all possible prospective scenarios can be tested retrospectively. Prospective scenarios that contain manual human steps (e.g., prompt clinician for symptom severity) cannot be tested retrospectively because the new prospectively collected data are not present in the retrospective dataset used in RetroGuide mode. Limited testing may be performed if the scenario can be decomposed into smaller sub-scenarios which are fully functional on retrospective data.

### HealthFlow External applications

We designed the HealthFlow system with several usage groups in mind. Our primary goal was to support the ability of a non-expert scenario requestor to passively understand the scenario logic and be able to review the logic. This also includes the ability to review any iterative changes to a scenario as it is being collaboratively authored by the requestor and the collaborating knowledge engineer. The *scenario-reviewer *role is mostly supported by the graphical nature of the scenario and the ability to use hierarchical arrangement of scenarios (i.e., a node may expand into multiple steps by using subflows). The secondary design goal was to also empower a non-expert requestor to even be the *scenario-author*, or to support very close requestor-knowledge engineer collaboration. To support a whole range of simple and complex functionality, we adopted an approach of several distinct HealthFlow *usage levels *(or complexity levels). This approach was inspired by historical development of the HealthFlow system, where we kept adding new, more complex, functionalities while also retaining their simpler versions for basic problems. Within this approach, we define three usage levels with increasing complexity: simple, advanced, and ultimate. A higher level can always utilize strategies and applications from all lower usage levels, and a given scenario may combine nodes (scenario steps) authored at different levels. During the last six years of developing the HealthFlow system, each usage level was populated with an initial set of external applications. However, each level can evolve and have additional capabilities added. The division into exactly three usage levels and functionality boundaries of each usage level are based on our analysis of existing HealthFlow scenarios, our interactions with scenario reviewers and authors, and system buy-in and training considerations (e.g., short training time and a favorable learning curve for simple problems, and non-expert users with the potential for more complex training and system use later).

The *simple level *uses EAs with a small number of parameters (zero, one, or two input parameters and one output parameter) which are easier to understand. A basic example of a simple EA is a lookup for patient gender using a step in a flowchart that contains an EA call of 'Patient_is_male()' and returns true if the patient is male, false if female, and null if gender information is not in the EHR. In a simple level, a separate EA is defined for each event type (total of 9 EAs, e.g., FindDiagnosis(ICDDxCode), FindLabResult(LabCode), FindVisit(AppointmentTypeCode)). Only a single event can be the result of a simple 'find event' instruction, and only a simple found/not found boolean output is available for scenario branching logic. All simple level EAs operate in a "strict pointer mode" which means that the hypothetical robot moves the current position pointer to the EHR position where its last step succeeded, or stays at the previous position if it failed. For example, a step using FindLabResult(LDL-cholesterol_code) after establishing the onset of lipid-lowering pharmacotherapy will return true if such follow-up test is found, and the current position pointer will move to a new EHR position. This behavior is similar to browsing and searching for events in a book; however, the pointer position can be manipulated by using "jumping" operations (e.g., JumpToLastEHREvent or JumpForwardXMonths).

The second, *advanced level *adds complexity to event finding operations by allowing greater granularity through more possible input and output parameters. In the advanced level, the current position pointer behavior can be modified to better fit user needs by enabling both strict and custom pointer modes. The custom pointer mode can restrict each find event operation with arbitrary time boundaries (usually defined in scenario variables). For example, FindCodedEvent with time boundaries of chronic kidney disease onset date to regular dialysis onset date: the outputs of advanced EAs are also extended and include all event properties such as event numeric value, coded value, or flag. Find event operation can return not just a single event, but also a set of multiple events, and the scenario can perform simple operations with these sets (e.g., use the count of events in the result set in branching logic such as if more than 3 dialysis events are found in the last 12 months since current or other arbitrary position). There is a total of 7 advanced EAs also with new types of applications added, such as EAs related to variables (AssignValueToVariable, IncreaseCounterVariable) and temporal EAs (EvaluateTwoTime-stampsDifferenceCriterion). Because of these additional capabilities, advanced level scenarios can be more complex to review for the requestor. The advanced usage level also offers the ability to export data generated within HealthFlow (using the HF-Export tool and CSV format) into external software packages (e.g., SAS or R). The export capacity is performed after scenario execution and could technically be part of any usage level, but is included in advanced level mainly for training purposes.

Thirdly, the *ultimate level *builds again on the previous levels and offers the ability to analyze averages and sums of numerical parameters of sets of events (e.g., average systolic blood pressure in a result set of a find operation). The ultimate version of the FindCodedEvent EA also supports the ability to use value sets derived from HealthFlow ontology and use terminology abstraction functionality. This enables it to use all children concepts (or only first degree children) with a particular relationship to a given term (most often the 'is_a' relationship). An example would be providing a concept code of a drug class (e.g., incretin mimetics) and let the HealthFlow ontology provide the enumeration of all current dispensable drugs in this drug class (e.g., exenatide, liraglutide, and taspoglutide). The ultimate level contains extended versions of some prior EAs and adds 4 new EAs and other model infrastructure dealing with retrospective to prospective transition (e.g., TriggerEventListener, RelevantEventListener). From a modeling perspective, the ultimate level also introduces the use of looping logic in a flowchart which may be required for some complex problems (e.g., determining onset of chronic kidney disease using laboratory criteria). Additional examples of EAs and their use can be found in previously published reports [33,37].

Finally, it is important to note that the HealthFlow system can be further extended to achieve functionality which is not covered by the external applications included in the three levels above. This is done by programming new, *problem-specific external applications*. Such new EAs may provide '*de nov*o' functionality such as: (1) access a legacy system or data stored within a different data representation model; (2) call an external web service; or (3) provide interface to advanced analytical features (e.g., call an external reasoning engine such as Neural Network, Fuzzy Logic, Hidden Markov Model framework, or other paradigm that may provide superior capabilities in some domain [e.g., dealing with reasoning under uncertainty]). Creation of such problem-specific EAs involves custom programming and requires collaboration with a programmer.

### An example use case

To illustrate HealthFlow use, it is perhaps best to operationalize it. Consider the following clinical scenario in osteoporosis care in women over the age of 67. Dr. Jones, a family medicine physician and a medical home initiative advocate, attends a conference where a case of a woman with repeated fractures after age 67 is described and recommended management options are emphasized (bone density testing and preventive administration of osteoporosis drugs). Upon returning from the conference, Dr. Jones wants to write a DSS module that would facilitate optimal care including the conference recommendations. He drafts an initial flowchart of this problem using a workflow process modeling tool. This tool enables Dr. Jones to model a sequence of analytical steps over EHR data and is easy to understand because it resembles a manual chart review. See figure 3 for the resulting initial flowchart.

**Figure 4:**
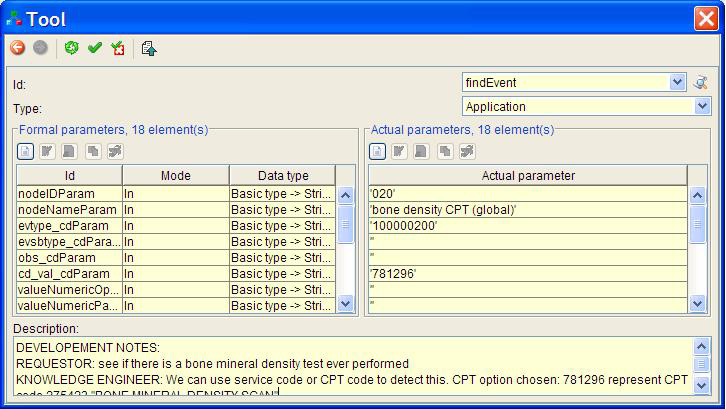
**External application example**. A workflow editor dialog box showing node properties window within a workflow editor. Details of the bone density node from osteoporosis scenario are shown. Ultimate level FindEvent external application was used in this case allowing the knowledge engineer the largest flexibility. Bottom parts of the figure shows an XPDL element where scenario-step specific communication of the requestor with the knowledge engineer can be achieved.

Mr. Clark, a collaborating knowledge engineer, receives the process flowchart in an email (XPDL file) from Dr. Jones and extends the nodes of the process flowchart with elements that enable execution of the flowchart. Figure 4 shows an example of a dialog box within Together Workflow Editor that specifies execution and parameters for an EA.

**Figure 5:**
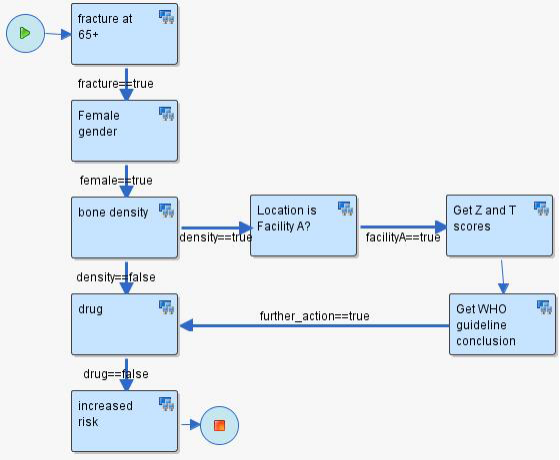
**Prospective version of the osteoporosis scenario**. Extended logic for flagship facility A. The added branch ensures that even if the bone mineral density test is present, the scenario will continue to evaluate the patien's medications if the density score indicate further action is needed. Notice that only the branches which make the scenario proceed need to be addressed. E.g., no branch for female = = false is needed.

To facilitate communication and consistency, the knowledge engineer preserves the structure of the flowchart (number of nodes, branches, and overall layout of the flowchart). The knowledge engineer then executes this extended process definition on a set of patients (e.g., 4000 females with a fracture) and emails back to Dr. Jones hyperlinks to web-based scenario retrospective execution reports (HealthFlow summary, detailed and IPV reports). Dr. Jones reviews the web-based reports and, based on a more detailed review of eight carefully selected patients, he identifies additional codes for bone density scores observed in the EHR of some of the patients. He decides to add a new process branch for a flagship family medicine center that is also collecting bone density Z and T scores in a structured EHR data entry form filled by medical assistants, which allows more specific decision logic within this branch. See figure 5 for the extended scenario. Several iterations of other process definition improvements occur, and Dr. Jones eventually concludes that the results of the retrospective fine-tuning of the process logic indicate that a meaningful and highly specific IT-intervention in a subset of osteoporosis patients is well justified. Dr. Jones uses the underlying process flowchart to share the analysis with his fellow physicians in the family practice clinic, who agree to a pilot deployment within their clinic. Dr. Jones sends the final executable scenario to the clinical decision support (CDS) team. The CDS team runs a transformation script against the process definition file which replaces all data retrieval process steps (which run against the data warehouse) with corresponding data retrieval calls that run against the production clinical data repository (CDR). The transformation script also changes the alerting/recommendation target from a retrospective execution report to a recommendation section within a patient dashboard screen in the EHR system. Since the logic prototyping was patient based (rather than population based), no other transformation for switching retrospective to prospective functionality is needed. During the pilot, colleagues of Dr. Jones can see new types of recommendations being generated, and they can click on the small view-logic-link next to the new recommendation. This view displays the same flowchart they were presented at their meeting with the highlighted route through the logic for a particular patient in question. The view offers them the ability to provide feedback linked to a specific node of the flowchart or to the flowchart as a whole.

### Other components of the HealthFlow system

#### Event Model

The event model used by the HealthFlow system is based on decomposition of EHR data into events described by Huff et al [22]. There are two event models supported: basic and extended. The three levels of EAs included in HealthFlow (simple, advanced, and ultimate) utilize the basic model. The extended model can accommodate any event structure, and in order to utilize it, custom EAs at the extended level have to be programmed. For example, we have piloted the use of the i2b2 event model.

**Figure 6:**
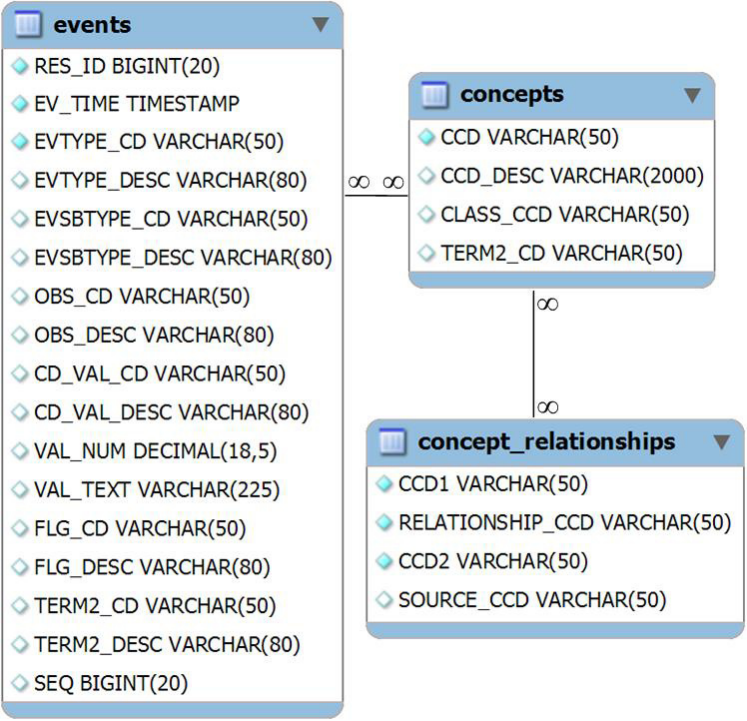
**HealthFlow event and ontology schema**. HealthFlow data model schema showing the event table linked to the ontology table. For events table examples, see table 2.

We further describe only the basic model. At the beginning of our workflow engine implementation project, our requirement was to create a simple event model that can cover 80% of the possible use cases, rather than an elaborate and comprehensive model. For example, the basic event model does not support the ability to recursively add additional event attributes outside of the provided basic set of event properties defined. The structure of the basic model has the following key event characteristics: patient_id, event date and timestamp, event type, event subtype, observation and coded value, value numeric, and value text. Additional characteristics are flag code, terminology2 code, and sequence_id. Each coded field has a corresponding code column and textual description column. An example of several observations (using only description fields) is provided in table 1. Figure 6 shows the event structure in an entity relationship diagram together with the ontology structure.

**Table 1:** Event schema example

EV_TIME (ev_time)	EVENT TYPE (evtype_desc)	SUBTYPE (evsbtype_desc)	OBSERVATION (obs_desc)	CODED VALUE (cd_val_desc)	FLAG (flg_desc)	VAL_NUM (val_num)	TERM2_CD (term2_cd)	TERM2_DESC (term2_desc)
1990-01-01 00:00:00.0	Birth event							
2046-04-23 00:00:00.0	Length of Stay					3		
2046-04-23 00:00:00.0	ICD-9-CM Diseases						72610	ROTATOR CUFF SYND NOS
2046-04-23 00:00:00.0	ICD-9-CM Procedures						8363	ROTATOR CUFF REPAIR
2046-04-23 00:00:00.0	CPT-4					2	J3010	Inj, fentanyl citrate
2046-04-23 00:00:00.0	CPT-4						29999	ARTHROSCOPY OF JOINT
2046-04-23 15:01:00.0	Clinical Text Data			Operative Report				
2046-04-23 15:23:00.0	Standard Lab Data	Lipid Profile	Cholesterol, Plasma Quant.		Higher Than Normal	327		
2046-04-23 15:21:00.0	Standard Lab Data	Urine Mi-croscopics	Epithelial Cells, Urine	Occasional				
2046-05-11 13:21:50.0	Problem Event	Diagnosis		Hyperlipidemia				
2046-08-12 11:12:13.0	Patient Order	Pharmacy order		Meperidine Hcl, 50 Mg/Ml, Ampul				
2047-01-18 10:55:01.0	Nurse Note						203.1.10.3.1.10.1.0	PURPOSEFUL MOVEMENT
2047-01-18 15:23:30.0	Inpatient Drug						3513816	ELECTROLYTES (NUTRILYTE) 42.9 ML, VIAL
2047-01-19 11:02:02.0	Discharged						43	ICU

Only selected sample events are shown. Dates are fictional and only textual meanings of codes are shown. The "ev_time" column shows the event time, the next four columns contain the basic four attributes (type, subtype, exam, coded value), followed by the flag and numeric value columns. Corresponding columns in the HealthFlow database schema (see figure 6) are shown in brackets in column headings. Only the event time and type attributes are required for all events. The last two "Term2" columns are used for storing external terminology original codes.

#### Ontology

Our workflow engine implementation, similar to other clinical informatics systems, needs a standard way of representing coded clinical concepts. Chosen representation approach has a high impact on semantic interoperability of individual scenario steps, and to ensure that we strive to utilize, as much as possible, existing and widely-spread terminologies. HealthFlow uses an internal native ontology that can accommodate standard established terminologies such as International Classification of Diseases (ICD) or Logical Observation Identifiers Names and Codes (LOINC), as well as local, proprietary ontologies. It supports any relationships among two concepts; however, the most important relationship is an "is_a" relationship. The HealthFlow ontology model also allows a concept to have multiple parents via the same or different relationship. For example, the ICD-9 code for 'prolonged depressive reaction' can have one parent of psychiatric disorder and also be part of the depression Healthcare Effectiveness and Data Information Set (HEDIS) value set. Another example would be the concept atenolol can have an 'is_a' relationship to a beta blocker concept and a 'has_indication' relationship to the hypertension concept. This enables the ontology to support the search for drugs that are indicated for a given disease (e.g., hypertension) as well as by drug class (e.g., beta blocker).

**Figure 7:**
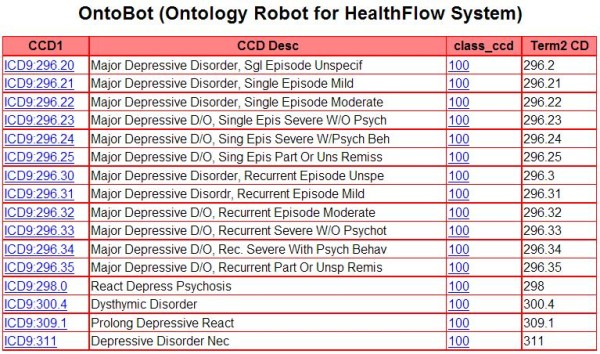
**Ontology browser**. Example showing HealthFlow internal ontology structure. This web-based applications allows the user to navigate concept children (HEDIS value set for depression diagnostic codes is shown). CCD abbreviation denotes a concept code.

In our current implementation, we have ICD-9-CM and Current Procedural Terminology (CPT) standard terminologies loaded. Included proprietary terminologies are local lab codes, local diagnostic codes, and local codes for medical specialties and vendor-supplied drug terminology. Additional relationships included for user convenience are HEDIS tables covering ICD-9 diagnostic value sets. Ontology relationships can be used only when ultimate EA sets are used. Our experience with requestors indicates that many of them are not interested in utilizing complex terminology structures and prefer directly enumerating concepts of interests for a given scenario step. Figure 7 shows the HealthFlow ontology web-based browser displaying first degree children of the is_member relationship of the HEDIS concept of AMM-A value set (Antidepressant Medication Management, table A: diagnostic codes to identify major depression).

## Results

### Current implementation at Marshfield Clinic

The workflow engine and the additional components of the HealthFlow system have been fully implemented at Marshfield Clinic (MC), and the system works in real time. We have created the necessary event listener (see figure 2) for MC's EHR (CattailsMD™). Both major components (RetroGuide as well as FlowGuide) are operational.

Under an approved RetroGuide Institutional Review Board (IRB) protocol, 40+ different scenarios were modeled by several analysts (see [Additional file 2] for examples) resulting in more than 360 execution reports being available in the RetroGuide project table. A RetroGuide scenario is usually tested on a cohort of 500 to 5,000 patients which is drawn from a larger sample of 75,000 patients. This larger de-identified cohort contains a total of 145 million coded EHR events. Table 2 shows an overview of selected event types such as diagnoses, procedures, laboratory results, and other structured EHR data. To demonstrate the ability to share decision support logic at a flowchart level, we have uploaded example scenarios to a decision support community wiki http://www.clinfowiki.org/wiki/index.php/HealthFlow.

**Table 2:** Coded EHR events statistics

EHR event type	Count
Lab	38,351,151
Diagnosis (ICD)	27,308,067
Procedure (CPT)	20,536,282
Vital signs	12,316,006
Textual report	12,205,115
Appointment	11,587,466
Prescription	7,522,419
Structured EHR value	7,323,129
Wellness	1,437,046
Electrocardiogram	1,122,398
Microbiology	703,451
Social History	302,698
Race and Ethnicity	78,654
Biopsy	28,317
Cancer History	18,557
Death (confirmed)	1,116

The table presents counts of events by type from the larger set of 75,000 patients used for sub-setting smaller testing cohorts for RetroGuide scenarios.

Under a separate FlowGuide IRB protocol, where real time EHR data is being used to model prospective scenarios, we are currently conducting a validation study of FlowGuide generated alerts in clinical domains of cholesterol management and rheumatoid arthritis. The current scenarios are designed to look at sub-populations of patients (e.g., patients with a history of LDL cholesterol above 190 mg/dL) and are triggered by the presence of a scheduled appointment event for the next day relative to the execution time. With the two deployed scenarios, the FlowGuide system daily processes between 150 and 400 patients and generates recommendations on 7.5% of them who are subject to the currently ongoing alert validation study.

### Previous case studies

In addition to the implementation at MC, several RetroGuide scenarios were executed against data from Intermountain Healthcare in the past (see [6] for full overview). The tested clinical domains included female Hodgkin's lymphoma patients, hypertension in diabetics, glucose management in intensive care unit patients, adverse drug events after use of narcotics, hepatitis C, osteoporosis, and cholesterol control. Three of those domains were also executed within RetroGuide using EHR data from Marshfield Clinic; so for three scenarios we demonstrated the ability to execute the same logic at two institutions.

### Formal, user-based evaluation

We have conducted a formal evaluation study of the HealthFlow flowchart-based modeling paradigm (retrospective mode only). In this evaluation, we used a group of 18 non-expert subjects in laboratory settings in a mixed method design. We quantitatively compared, by measuring their performance on a test, the ability of subjects to solve query problems using flowcharts against a traditional code-based structured query language (SQL) representation. The test included 9 task questions and 5 choice questions. In a task question, the subject had to create a solution for a given search task. For example, task T4 was "Find all patients who had at least 2 creatinine lab results flagged as too high." In a choice question that evaluated the ability to review a solution, the subject was shown an initial solved problem and was asked to choose from one of the three presented solutions which correctly extends the initial solution with additional query elements. In another example, the extended task C5 was "Find all patients who experienced the initially mentioned adverse drug event (naloxone and transfer to ICU within 6 hours) and also had a record of sleep apnea diagnosis prior to this adverse drug event."

In a follow-up qualitative study, we investigated technology acceptance of the flowchart-based representation paradigm using the UTAUT model (Unified Theory of Acceptance and Use of Technology) [38]. A detailed description of the design and results of this evaluation has been published separately [6]. The quantitative results indicated that non-experts achieved significantly higher scores in problem solving using flowcharts-based technology as compared to a purely code-based technology. The flowchart-based technology had on average 4.8 points higher test scores (SD = 1.8, CI 3.3-5.4; p < 0.0001) on a scale of 0-14 points. The qualitative follow-up study results showed that 94% of the subjects preferred flowcharts to code because it was easier to learn, it better supported temporal tasks, and it seemed to be a more logical modeling paradigm.

### Architectural evaluation

In addition to this laboratory evaluation, we also looked at the workflow engine implementation in comparison to other decision support architectures. We used a previously published evaluation framework [15] (referred to as CDS-EF = Clinical Decision Support architecture - Evaluation Framework) for this architectural evaluation. This evaluation framework has been previously applied to Arden Syntax, GLIF, SAGE [39], SEBASTIAN [40], and SANDS (service-oriented architecture for decision support [15,41]). We briefly comment on each of the CDS-EF elements of (1) feature determination, (2) existence and use, (3) utility, and (4) coverage.

(*1) Feature determination: *HealthFlow was developed with numerous CDS-EF's desirable features in mind, and it evaluates favorable against them. Specifically, HealthFlow:

• Is *shareable*, because it is based on a cross-industry workflow standard.

• Maintains *DSS content separate from EHR code *by using a workflow management layer running on top of an EHR system.

• *Content integrates into workflow *by directly utilizing WfMC's model for modeling participants roles and building on the concept of tasks and worklist handlers [16].

• *Supports event driven CDS *through modeling appropriate triggers in a process definition and sending corresponding EHR system event data to the workflow engine.

• *Supports non-event driven CDS *through pull requests originating from non-event-driven systems, such as dashboards or reporting applications.

• *Avoids vocabulary issues *by implementing a flexible ontology layer and also through the ability to call external terminology services via external applications.

• *Enables composition of rules *because XPDL language allows subflows where a given piece of logic can be re-used in multiple scenarios

• *Allows black-box services *where a scenario can invoke via multiple mechanism (most likely via a web-service call) an external inference service without exposing its underlying inner logic to the underlying WfMS

• *Has free choice of programming language *since scenario-referenced external applications can be written in any language if they comply with the XPDL-defined standard interfaces.

*(2) Existence and use: *CDS-EF proposes a four level spectrum for evaluating the existence and use axis ranging from theoretical discussions (level 1) through widespread adoption (level 4). With RetroGuide, we achieved level 3 - advanced prototypes that demonstrate sharing of decision support content across sites. We were able to demonstrate running the same XPDL logic against data from two healthcare institutions (Intermountain Healthcare and Marshfield Clinic). However, when we consider workflow technology-based process modeling as a general technology, it also reaches level 4 (implementation in commercial EHR systems) when we include examples from Siemens [14] and other case studies [42].

*(3) Utility: *The CDS-EF distinguished two faces of utility: clinical utility and functional utility. Clinical utility is the ability to use a decision support architecture to deliver clinically relevant outputs. HealthFlow's clinical utility is determined by the clinical utility of the use cases implemented in it. To date, implemented use cases were in the domains of retrospective prototyping of decision support logic, identifying opportunities for interventions that would improve compliance with healthcare quality improvement measures, modeling enrollment or outcome-tracking logic for clinical trials, and finally validation of prospectively generated decision support alerts in four clinical domains.

Functional utility is the ability of an architecture to support a variety of different kinds of clinical decision support. Selected functional utility aspects of our workflow engine implementation are:

• *Developer *(non restrictive technical architecture): XPDL is an open, non-proprietary and evolving standard which covers a robust set of workflow patterns.

• *User *(wide range of possible DSS target users): a HealthFlow scenario can include steps for various roles ranging from clinician, administrator, as well as patient (e.g., via personal health record)

• *Information source *(retrieval of data from any system): XPDL's ability to call external systems and applications ensures ability to obtain data from any knowledge or data source.

• *Clinical purpose *(support for full range of potential use cases): processes defined in a workflow engine are not restricted to a particular use case and, depending on the host EHR, can complement diagnostic as well as therapeutic scenarios for a single patient or for a population of patients, and can be delivered as a passive information display as well as active interaction with a clinician at the point of care

The described workflow engine implementation evaluates favorably against the remaining functional utility parameters of supporting several inference types, ability to combine scenarios via subflows, and can be implemented in various business models.

*(4) Coverage: *The final element of the CDS-EF is the ability of a decision support architecture to encode clinical knowledge in comparison to other approaches. This final evaluation element is based on a taxonomy derived from a set of 7,120 rules [35] implemented at some point at Partners healthcare system. The taxonomy represents a set of functionality features, which many recent DSS architectures such as SANDS [41], use as a minimum required functionality set. Our HealthFlow DSS architecture was specifically extended to be able to support 100% of possible modalities within the four categories in the taxonomy. However, it is important to note, that this compliance assumes that a target EHR supports all aspects of the HealthFlow active and passive EHR interaction model (see figure 2).

## Discussion

In this paper, we have described our implementation of workflow technology for the purpose of clinical decision support. Our implementation addresses the problem of retrospective testing and user-friendly flowchart representation of the clinical logic. We have described the use of this framework on multiple case studies at two institutions and results of two evaluations. We will first look at advantages and disadvantages of our architecture in general, as well as the currently used software (Together workflow editor and engine). Finally, we will discuss the relationship of workflow technology to query systems and look at possible future developments and long-term relationship of workflow technology to decision support engines.

### Strengths and limitations of our architecture

Unlike many existing decision support frameworks that introduce healthcare specific knowledge representation standards (for example SAGE [39], GLIF [3], ProForma [43] or SANDS [41]), our implementation relies on a cross-industry workflow technology architecture and open, well-defined XPDL standard. We see this as a major advantage since the workflow technology software tools may improve in time based on usage in other industries such as manufacturing, shipping, banking, and insurance industries. Future enhancement to workflow editors, engines, and other tools are thus not driven by healthcare alone. The use of a standard workflow representation language (XPDL) also ensures that clinical scenario logic developed at one healthcare institution can be used directly (or with little modification) at another healthcare institution. Moreover, by utilizing a standard workflow definition language, it is possible to use different underlying workflow engines or editors at different institutions.

One limitation of our approach is the readability of very complex scenarios. While every new technology offers some advantages and simplifies a view of complex problems, there is an absolute limit to any such simplification. Flowchart-based graphical representation of very complex algorithms will, in extreme cases, naturally result in a very complex flowchart. Hence, the advantage of transparency of a graphical format may not be apparent. This limitation can be alleviated by using a hierarchical arrangement of scenarios where a node in a higher lever flowchart expands into a sub-flow which may consist of multiple steps. Long and complex scenarios can thus be reduced to fewer, high-level flowchart nodes that group related analytical steps. The complexity problem can also be addressed by shifting some of the logic away from a flowchart into a single rule-based node, that calls a comprehensive rule-base or other expert system. In many implemented scenarios, however, we often chose to represent as much logic as possible using the flowchart and XPDL constructs (e.g., interim decision state nodes and strict use of transition conditions) to preserve a clinician-friendly scenario review.

### Strengths and limitations of currently used workflow suite

Workflow suite developed by Together was the leading open-source workflow software available when we started our workflow technology implementation project, and it continues to be the most robust, XPDL-compliant workflow suite today. For the editor component, there are two main advantages. The first is the ability to customize functionality for a particular use case because the product is open-source and the code is available to download. This enabled us to implement the flowchart display feature on HealthFlow summary report by calling a flowchart rendering sub-component into our visualization component. The second advantage is an implementation of the flowchart notation that does not require the use of a separate join and split node for flowchart branches, [44] which makes the resulting flowcharts simpler to review. A disadvantage of the editor is implementation of the XPDL 1.0 standard instead of the most recent 2.1 version, although this is on the project future roadmap. For user-friendly editing, the editor also lacks ability to change size of the flowchart boxes, use custom colors and fonts, annotate flowchart with additional notes visible in the graphical flowchart, and streamline creation of subflows by selecting a set of nodes in the existing flowchart.

As for the engine component, a major advantage is the ability to deploy the Together Workflow Engine on multiple operating systems (Windows and Linux) and interact with it via several platforms (stand-alone, within JBOSS container, or as a set of integrated web-services). A key disadvantage, in general, is the focus of the engine on a set of configurable and programmable components rather than one integrated and compact product. For example, to link the event listener with the engine, we had to create two small pieces of software that materialize the link between the engine with our Event listener (see figure 2) for trigger steps and listen steps for FlowGuide. Also, there are no robust tools provided for exporting or detailed analysis of the workflow logs of past processes, which we achieved by analyzing the internal database of the Together workflow engine. Finally, we have also encountered clinical scenarios where we wanted to enable multiple entry points into the decision logic, which is functionality possible within some medicine-specific guideline standards (e.g., GLIF). An XPDL process model can formally have only one starting point; however, it is possible to solve the problem with one additional dummy workflow node that is connected via an additional logic of trigger conditions, and the logic flow is thus routed to the desired later start point in the flowchart. The resulting flowchart is, however, somewhat cluttered with extra transitions resulting from such a solution.

### Relationship to query systems

Although HealthFlow's main focus is on deployment of clinical logic with impact at the point of care, it can also be used as a query technology - using strictly the retrospective mode and only the RetroGuide component. RetroGuide could then be compared to cohort estimation (or query building) tools such as i2b2's query component [45] or STRIDE (a proprietary patient cohort discovery tool developed at Stanford university [46]). The key difference with such tools is that RetroGuide execution strategy is to process each patient separately, which mimics how a decision support module works (on a single patient, instead of a population). This factor is important when some complex query elements are considered. The problem can be described as *inter-element parameter passing *and the ability to create and manipulate *interim constructs *(e.g., LDL cholesterol value prior treatment). Any query tool decomposes a query problem into simpler *query elements*. For example, a query of finding female patients with fractures would be decomposed into two elements: (1) female gender and (2) fracture event. In this example, query elements are independent of each other. However, computation of certain temporal problems or relative value comparisons makes some query elements depend upon results of prior query elements. For example, a query targeted at regular thyroid stimulating hormone (TSH) screening compliance in patients on levothyroxine requires determination of levothyroxine therapy onset (defined as first two levothyroxine pharmacy pick-ups within a 6 month window), and passing of this time parameter to later evaluation for regular TSH lab tests in the following 12-month time window until the patient's present visit. An additional problem with the element dependency phenomenon is that the dependency is usually on a patient level, rather than on a population level (which is a problem for query tools that ultimately translate the query into a single SQL-based, populational query, and which may lack support user-defined interim constructs).

An example of relative value comparison is the problem of detecting a clinically significant response to depression therapy: "find patients with initial PHQ-9 score of 15 or more, who in subsequent testing after a therapeutic intervention lowered their score by at least 50%" (parameter passed: initial PHQ-9 score). Notice that the problem is different for different patients (e.g., 16 reduced to 8, 24 reduced to 12) and it can only be solved with a somewhat complex population-based SQL query using interim tables and is not easily solvable with most SQL-based query tools [24]. Another temporal example is to "find patients who had a bone mineral density test prior to their first hip fracture episode" (parameter passed: date of first hip fracture). Ability to represent temporal logic is often listed in desired features of many query tools; however, it can be considered a special case of what we define in a more general sense as inter-element parameter passing.

Our workflow technology implementation, which builds on a procedural and flowchart-based paradigm rather than a declarative language, would be able to solve such problems because (1) it can utilize variables for inter-elements parameter passing (e.g., initial PHQ-9 score), and (2) it has a built-in single patient execution level. Query tools utilizing mostly SQL representation logic can only solve such problems when an *interim construct *feature is present. However, most tools do not support such pre-processing within their graphical user interface (GUI), and it needs to be done by a programmer (see Deshmukh's evaluation of i2b2 search tool for examples of pre-processing in i2b2 [24]).

Another unique aspect of the workflow technology process-based approach can be described as *GUI-unrestricted creativity*. In most query building tools, the user usually constructs a query by combining a fixed set of query features built into a particular user interface, such as business intelligence authoring tools or Microsoft Access query wizard. Such user interface can also involve additional query metaphors such as Venn diagrams or PubMed-like advanced search combining interim search steps with logical operators (e.g., (#4 OR #8) AND #13). The set of graphical GUI features, or widgets, is always finite and the non-expert user is restricted to this set of features within a GUI-based *query building tool*. A chosen graphical metaphor of the query building tool may not support all necessary query elements. (e.g., within patient aggregation combined with other query elements: average systolic blood pressure 5 years prior to start of hypertension pharmacotherapy). This is in contrast to a *direct authorship of the query code *method employed by an experienced programmer who uses the same underlying query language as the GUI tool, however, is able to overcome some of the restricted widget set problems. In many tools there is an option for dual or combined interface for query authoring, so the difference may not be apparent in basic query tasks. However, the advanced nature of some tasks created via direct code authorship often breaks the link between these two corresponding representations (graphical and code representation) paradigms. Whereas it is almost always possible to transform GUI-authored changes into the code form, the tool (e.g., business intelligence tools) may not always support the ability to transform changes made directly in the code back to the graphical user interface representation form.

The workflow process, flowchart-like modeling paradigm is trying to retain a user friendly look for non-experts and still offer the unrestricted creativity of a direct code authorship, using a hybrid form combining both aspects of graphical and code paradigm. In a traditional search tool GUI, a new solution opens with a screen with a limited number of widgets or language constructs. Whereas, in creating a representation of a new clinical logic problem, he or she is facing a blank-sheet-of-paper paradigm as opposed to a screen with a limited set of buttons or features.

### Future directions in workflow technology and relationship to decision support engines

Workflow technology suites have evolved substantially since the creation of the Workflow Management coalition family of workflow standards. We expect this trend of feature improvement of workflow suites to continue, and we plan to incorporate such improvements into our implementation. For example, several prior scenario display disadvantages (such as direct display of transition conditions in the flowchart, improved storage of flowchart layout, and several new interface customizations) were addressed in a recent upgrade from version 2 to version 3 of Together Workflow Editor. Besides incorporating further improvement in workflow technology, our future work is focused on demonstrating recursion and loops functionality, better utilization of workflow-engine generated process logs in addition to existing HealthFlow summary and detailed reports, providing more options for interacting with the EHR system in the prospective execution mode, wrapping some of HealthFlow's capabilities into a web service accepting an HL7's Clinical Document Architecture (CDA) EHR data, demonstrating portability of the XPDL format with additional vendors, and additional cognitive evaluation of the flowchart-based problem representation paradigm.

From looking at the development of healthcare-specific decision support engines and workflow technology, it is also clear that these two technologies overlap significantly [10]. Almost all healthcare-specific decision support engines implement, to some extent, workflow features; for example, representation of roles and individuals within an organization, or worklist handler functionality. Conversely, most workflow engines suites or specific site-implementations increasingly incorporate more decision support oriented features; for example, seamless integration of a rule based engine. Both of these trends are naturally motivated by the simple fact that both reasoning capabilities (support for workflow as well as for decision making trade-offs or complexities) are needed in healthcare. Vendors of both technologies are well aware of this overlap, and in the future we can expect more feature-rich and integrated engines. From a software terminology perspective, we can perhaps even expect a new software system term, such as *comprehensive reasoning engine*, which will explicitly describe a combined decision support and workflow engine. Another possible future scenario is that one technology may dominate or even completely subsume the other.

#### Comparison to other decision support formalisms

The main focus of our article has been on describing an open-source workflow engine implementation; however, because of the application domain of decision support, we briefly comment on the relationship to some healthcare-specific decision support or executable guidelines formalisms [47]. For a detailed discussion of this topic, we refer the reader to Peleg's analysis in a book chapter titled 'Guidelines and workflow models' [10] of recent DSS books and recent guidelines review studies [12,48,49]. Peleg [10] defines a subgroup of decision support formalisms referred to as task-network models, which include a graphical metaphor for representing clinical logic. This subgroup is most relevant to a workflow engine implementation relying on an XPDL standard which also natively includes a graphical flowchart layer. Peleg includes in this group the following representation formats: Asbru [50], closely related format group consisting of EON, PRODIGY [51] and GLIF, GUIDE/NewGUIDE [52], SAGE, ProForma [53] and GLARE [54]. Many of these formats have considerable strengths in modeling workflow and offer more sophisticated medicine-specific modeling constructs. We briefly comment on differences and the relationship of our HealthFlow system implementation to some of these formats. Compared with Asbru, HealthFlow does not include any tools for transforming textual guidelines into executable form. Compared with the GLIF group of formats, HealthFlow does not distinguish between the two flowchart types of action map (with physical steps) and decision map (reasoning steps) and lack several other medicine-specific reasoning extensions of those formats. GUIDE/NewGuide format is probably the most related to HealthFlow because it includes a workflow management system from Oracle [55]. In contrast to ProForma, HealthFlow lacks constructs for rule-in, rule-out logic [56] that was later adopted by many other medicine-specific formats. Unlike many academic-driven formats, ProForma evolved into a commercially supported platform [57] which is also the case with workflow engine technology. Finally, in contrast to numerous medicine-specific formats, the XPDL language and our HealthFlow workflow engine implementation does not include any built-in support for uncertainty. The existing implementation operates strictly on coded EHR concepts where all utilized facts are considered fully valid and only in cases where significant data unreliability is suspected by scenario authors, the scenario logic may include some additional information validation steps (e.g., minimum of two billing diagnostic codes). However, in some scenarios, we are considering inclusion of steps with natural language processing of free-text medical reports. Such scenarios would obviously have to deal with some uncertainty measure and the HealthFlow extensibility feature via new EAs or external engines would most likely be utilized where XPDL-based flowchart paradigm would be insufficient. However, some approach to uncertainty would also have to be adopted by the host EHR system, and currently there is only limited support for this in our institution's retrospective data or EHR system (e.g., uncertainty constructs within the problem list management or treatment planning).

## Conclusion

The described software enables modeling and execution of clinical decision support problems and addresses two important challenges of retrospective testing and user-friendly flowchart representation. Additional advantages are extensibility via using external applications, native single patient execution, ability to handle temporal logic, ability to define and later use interim constructs, and code-like, unrestricted logic creation paradigm. Using a cross-industry workflow technology and open source and freely available components, we were able to create a decision support platform which evaluates well against an established clinical decision support architecture evaluation framework.

## Availability and requirements

The workflow engine technology implementation described above can be implemented at other institutions. The article describes fully the necessary architecture and software components. The utilized workflow editor and engine are open-source and free for non-commercial use (GNU General Public License (GPL)). The Together Workflow Engine (Community Edition) is freely available for download at http://sourceforge.net/projects/sharkwf under the GPL v3 license. The Together Workflow Editor is also freely available for download at http://sourceforge.net/projects/jawe under the GPL v3 license. Complete source code is also available for download for both components. Installation of the workflow editor and review of scenarios is a simple task on any Linux, Windows, or Mac computer with Java Development Kit (JDK). A no-installation editor edition (Java webstart) is also available. Installation and configuration of the workflow engine requires some technical experience and can be done on a Linux or Microsoft Windows server or a workstation with JDK version 5.0 (1.5.0.11 or above), JBoss Application Server 4.2.x GA, and a database (such as MySQL Community Server version 5.1 or above). In our installation, we use the JBoss implementation of the engine, but it can also run on other J2EE platforms (e.g., TomCat, WebLogic) or as a stand-alone Java application, and it can be accessed via several possible APIs (application programming interfaces). Workflow engine administration is performed via a Java administration tool or a web-based application. Together workflow engine can also be linked with an existing LDAP (Lightweight Directory Access Protocol) server for user authentication, user groups and organizational structure. Libraries and documentation for the HealthFlow specific components are freely available at http://code.google.com/p/healthflow under the GNU General Public License. Healthcare institutions implementing the above-described framework may use the provided examples of decision support logic modules (see [Additional file 2] and project website) or create new modules that specifically address their local decision support needs or more adequately integrate with their local organizational context and clinical user roles.

## Competing interests

The authors declare that they have no competing interests.

## Authors' contributions

VH conceived the study and planned the initial architecture. VH and LVR were involved in configuring the workflow engine and editor and development of site specific components. All of the authors participated in the study design, coordination, and in preparing the written manuscript.

## Pre-publication history

The pre-publication history for this paper can be accessed here:

http://www.biomedcentral.com/1471-2288/11/43/prepub

## Supplementary Material

Additional file 1 **Additional XPDL standard schema diagrams**. Additional file 1 contains detailed diagrams of the XML Process Definition Language (XPDL) as defined by the Workflow Management Coalition standard. Click here for file

Additional file 2 **Additional examples of HealthFlow Scenarios**. Additional file 2 contains six additional examples of HealthFlow scenarios, including the rheumatoid arthritis scenario deployed currently in production at Marshfield Clinic for the currently ongoing HealthFlow validation study. Click here for file
