# Supplementary material for: Implementation of workflow engine technology to deliver basic clinical decision support functionality
Source: BMC Med Res Methodol. 2011 Apr 10;11:43. doi: 10.1186/1471-2288-11-43 (PMC3079703; doi:10.1186/1471-2288-11-43)

**Appendix A: XML Process definition language**

Note:Blue nodes highlight the most important element in the diagram

The key element of an XPDL file is the package element. It contains one or several individual processes (e.g., Diabetes management)


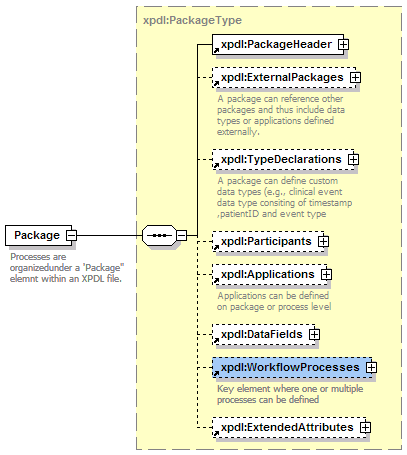


A workflow process consists of one or multiple activities (e.g., Determine diabetes onset, Check current diabetes medication, Obtain most recent diabetes lab results (HbA1c)).


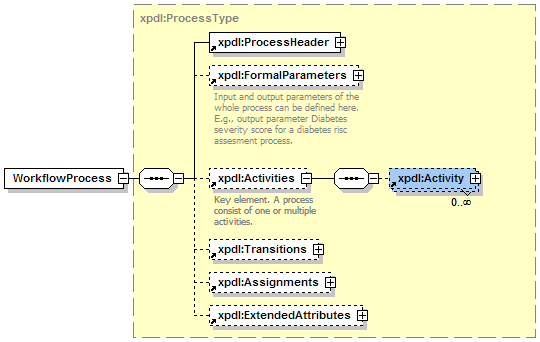


There are many different types of activities defined. The most important type is activity of a ‘Task’ implementation type. (e.g., EHRGetLabData(PatientID, LabTestCode), or EHRRegisterAlertMessage(PatientID,BloodPressureNotAtGoalMessage)


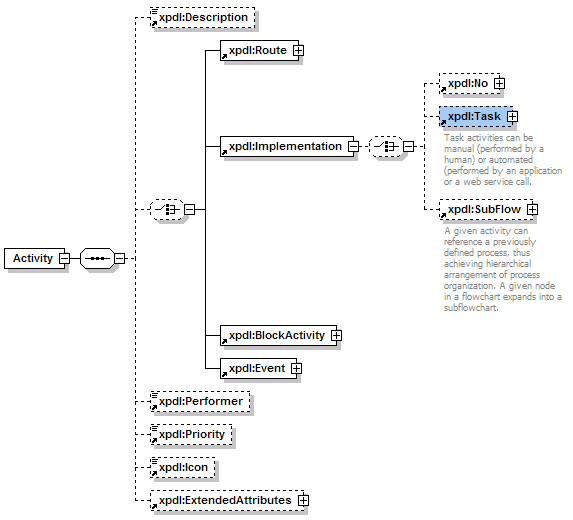

Supplement: Additional file 1 — Additional XPDL standard schema diagrams. Additional file 1 contains detailed diagrams of the XML Process Definition Language (XPDL) as defined by the Workflow Management Coalition standard. [file 1471-2288-11-43-S1.DOC]
