# Supplementary material for: Implementation of workflow engine technology to deliver basic clinical decision support functionality
Source: BMC Med Res Methodol. 2011 Apr 10;11:43. doi: 10.1186/1471-2288-11-43 (PMC3079703; doi:10.1186/1471-2288-11-43)

Appendix B: Additional HealthFlow flowcharts

# Diabetes

HealthFlow can be used to model phenotype determination – either in RetroGuide retrospectively or in FlowGuide – prospectively – for example prompt to enroll into a RCT every diabetic as they visit a facility. Pink nodes a routing nodes – do not contain any external application execution.


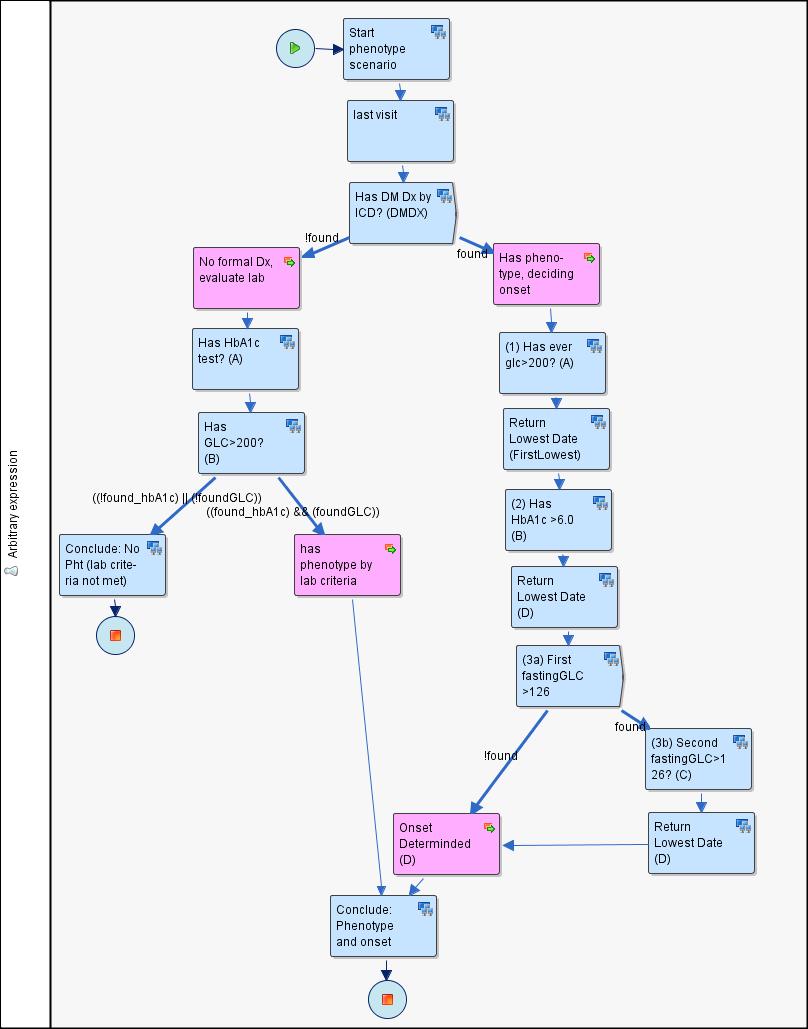


# Hypertension

 Example clinical decision support logic. The scenario can be executed prospectively as well as retrospectively (there is no user interaction, just working with current or past EHR events).


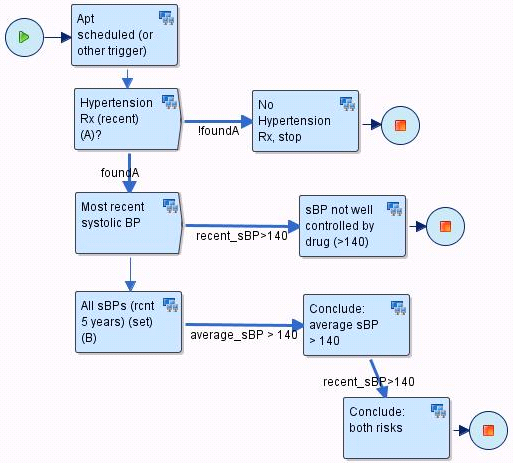


# Primary Care drug triggered lab monitoring 1

This scenario is inspired by primary clinician’s standing instruction to his medical assistant.


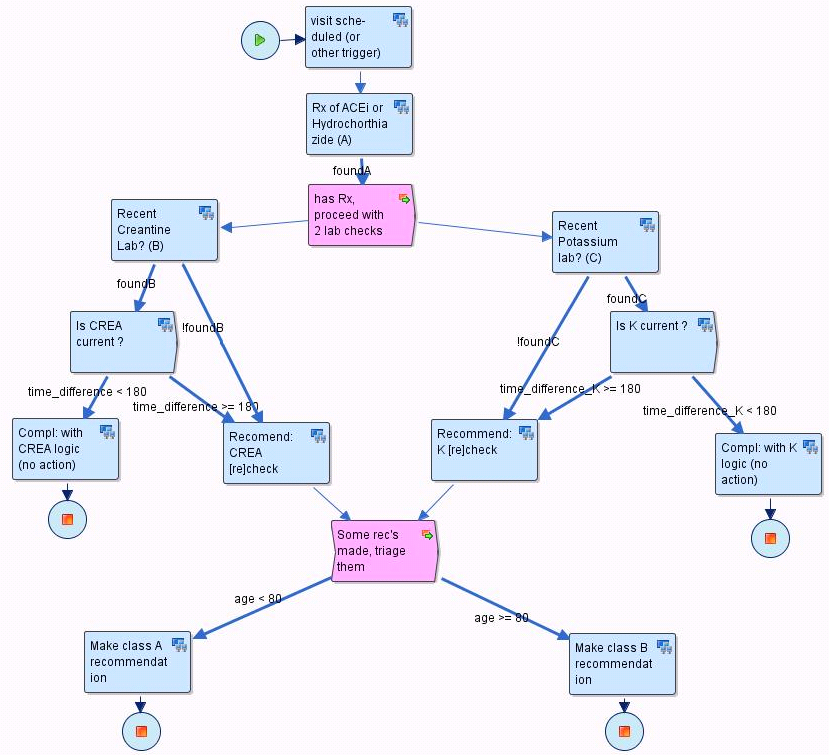


# Primary Care drug triggered lab monitoring 2


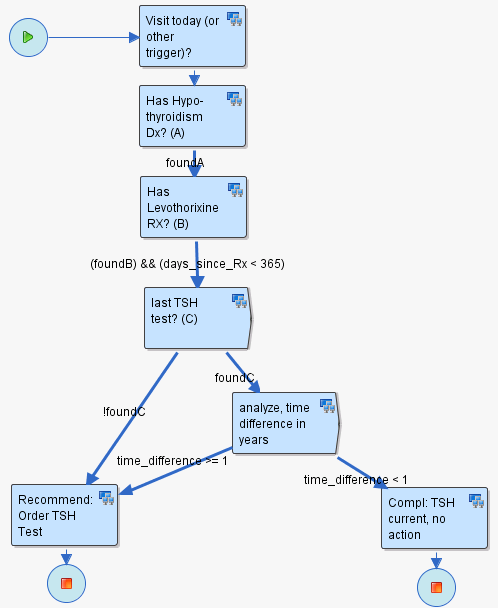


# Referral to genetic counseling

Red nodes represent a link to a sub flowchart with additional logic. In most EHR systems, there is only limited coded family history data available, however the scenario can still evaluate the score using patient history data.


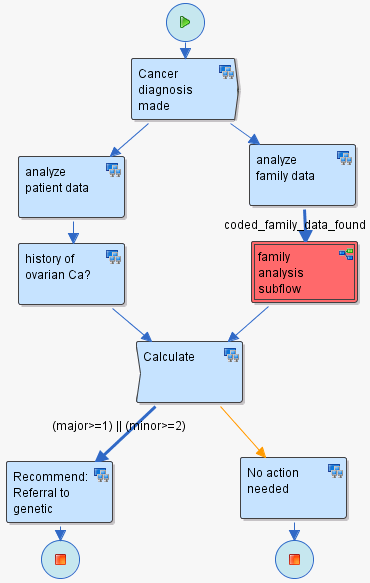


# Two rules in rheumatoid arthritis domain (Tb screen, PneumoVax)

FlowGuide prospective scenario monitoring two rule sets in Rheumatoid arthritis clinical domain. Purple nodes are dummy routing nodes and do not contain any executable logic.


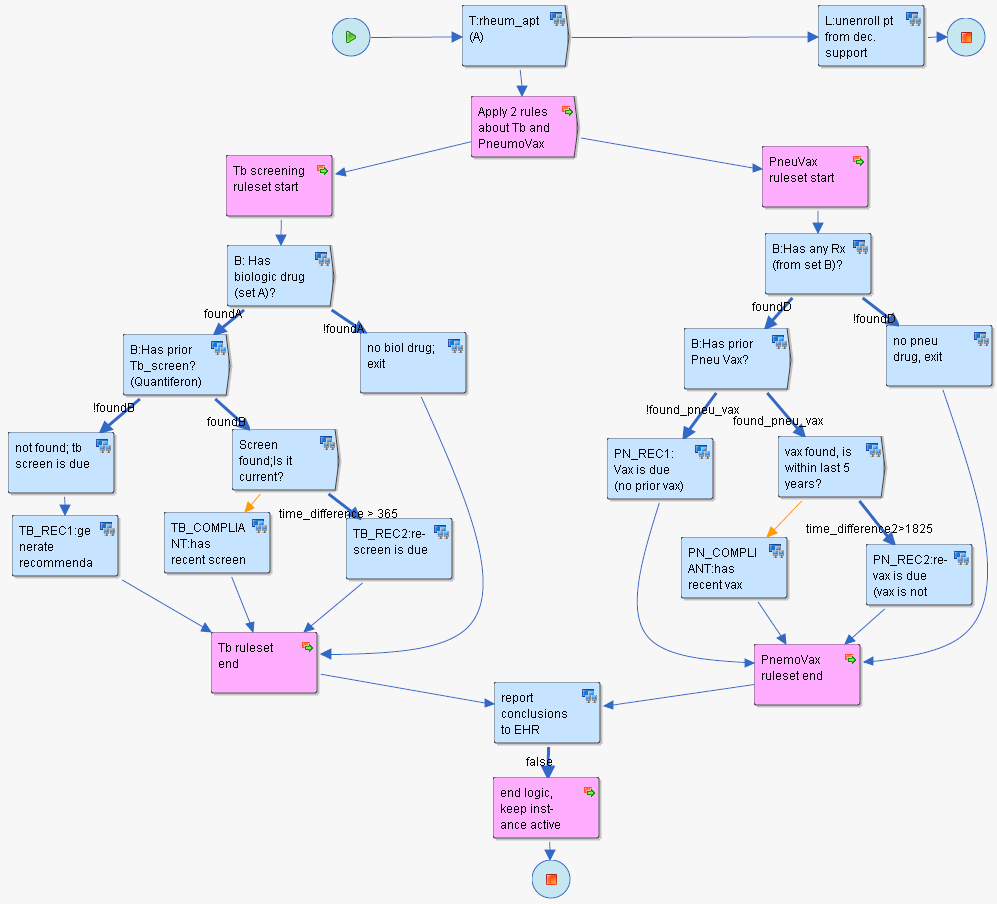

Supplement: Additional file 2 — Additional examples of HealthFlow Scenarios. Additional file 2 contains six additional examples of HealthFlow scenarios, including the rheumatoid arthritis scenario deployed currently in production at Marshfield Clinic for the currently ongoing HealthFlow validation study. [file 1471-2288-11-43-S2.DOC]
